# Supplementary material for: From sequence to enzyme mechanism using multi-label machine learning
Source: BMC Bioinformatics. 2014 May 19;15:150. doi: 10.1186/1471-2105-15-150 (PMC4229970; doi:10.1186/1471-2105-15-150)
Supplement: Additional file 2 — Java code of ml2db. Additional file ml2db_code.tar.gz contains the Java source code to run the multi-label machine learning experiments and save the results to database. The code’s Javadoc is included. [file 1471-2105-15-150-S2.zip › additional file 2/ml2db/ml2db/doc/main/ml2db/mulan/learn/MulanLearner.html]

MulanLearner


JavaScript is disabled on your browser.


- Overview
- Package
- Class
- Use
- Tree
- Deprecated
- Index
- Help

- Prev Class
- Next Class

- Frames
- No Frames

- All Classes

- Summary:
- Nested |
- Field |
- Constr |
- Method

- Detail:
- Field |
- Constr |
- Method


main.ml2db.mulan.learn

## Class MulanLearner

- java.lang.Object
- - main.ml2db.mulan.learn.MulanLearner

- All Implemented Interfaces:
  :   java.io.Serializable

  ---

    

  ```
  public class MulanLearner
  extends java.lang.Object
  implements java.io.Serializable
  ```

  Containing a Mulan learner and its name, that is: the name of the
  MultiLabelLearnerBase plus the name of the algorithm within

  Version:
  :   1 Sep 2010

  Author:
  :   Luna De Ferrari ldf(at)st-andrews.ac.uk

  See Also:
  :   Serialized Form

- - ### Constructor Summary

    Constructors

    Constructor and Description

    `MulanLearner(java.lang.String learnerBaseType,
    java.lang.String learnerType)`

    Incomplete constructor: it is essential to then set a learner of the same
    type specified!

    `MulanLearner(java.lang.String learnerBaseType,
    java.lang.String learnerType,
    mulan.classifier.MultiLabelLearnerBase learner)`
  - ### Method Summary

    Methods

    | Modifier and Type | Method and Description |
    | --- | --- |
    | `mulan.classifier.MultiLabelLearner` | `getLearner()` |
    | `java.lang.String` | `getLearnerBaseType()` |
    | `java.lang.String` | `getLearnerFullName()` Get the full type of a learner: the name of the multi-label learner base/wrapper plus the weka or mulan algorithm/classifier name |
    | `java.lang.String` | `getLearnerType()` |
    | `static java.lang.String` | `getName(weka.classifiers.Classifier classifier)` |
    | `static java.lang.String` | `getName(mulan.classifier.MultiLabelLearner learner)` |
    | `static java.lang.String` | `getName(java.lang.String name)` remove the object number from the class name |
    | `void` | `setLearner(mulan.classifier.MultiLabelLearner learner)` |
    | `java.lang.String` | `toString()` |

    - ### Methods inherited from class java.lang.Object

      `equals, getClass, hashCode, notify, notifyAll, wait, wait, wait`

- - ### Constructor Detail


    - #### MulanLearner

      ```
      public MulanLearner(java.lang.String learnerBaseType,
                  java.lang.String learnerType)
      ```

      Incomplete constructor: it is essential to then set a learner of the same
      type specified!

      Parameters:
      :   `learnerType` -
      :   `learnerBaseType` -


    - #### MulanLearner

      ```
      public MulanLearner(java.lang.String learnerBaseType,
                  java.lang.String learnerType,
                  mulan.classifier.MultiLabelLearnerBase learner)
      ```
  - ### Method Detail


    - #### getLearner

      ```
      public mulan.classifier.MultiLabelLearner getLearner()
      ```


    - #### getLearnerBaseType

      ```
      public java.lang.String getLearnerBaseType()
      ```


    - #### getLearnerFullName

      ```
      public java.lang.String getLearnerFullName()
      ```

      Get the full type of a learner: the name of the multi-label learner
      base/wrapper plus the weka or mulan algorithm/classifier name


    - #### getLearnerType

      ```
      public java.lang.String getLearnerType()
      ```


    - #### setLearner

      ```
      public void setLearner(mulan.classifier.MultiLabelLearner learner)
      ```

      Parameters:
      :   `learner` - m\_learner to set


    - #### toString

      ```
      public java.lang.String toString()
      ```

      **Overrides:**
      :   `toString` in class `java.lang.Object`


    - #### getName

      ```
      public static java.lang.String getName(weka.classifiers.Classifier classifier)
      ```


    - #### getName

      ```
      public static java.lang.String getName(mulan.classifier.MultiLabelLearner learner)
      ```


    - #### getName

      ```
      public static java.lang.String getName(java.lang.String name)
      ```

      remove the object number from the class name

      Parameters:
      :   `name` - a class name containing or not the at symbol

      Returns:
      :   the class name without the string following the at symbol
          (without the object number)


- Overview
- Package
- Class
- Use
- Tree
- Deprecated
- Index
- Help

- Prev Class
- Next Class

- Frames
- No Frames

- All Classes

- Summary:
- Nested |
- Field |
- Constr |
- Method

- Detail:
- Field |
- Constr |
- Method
